# The Influence of the Timing of Cyclic Load Application on Cardiac Cell Contraction

**DOI:** 10.3389/fphys.2018.00917

**Published:** 2018-07-18

**Authors:** Lior Debbi, Stavit Drori, Shelly Tzlil

**Affiliations:** Faculty of Mechanical Engineering, Technion – Israel Institute of Technology, Haifa, Israel

**Keywords:** stretch device, cardiac single cell mechanics, cyclic stretch, mechanical stimulation, traction force imaging, electromechanical coupling, biomechanics, electrical stimulation

## Abstract

Cardiac cells are subjected to mechanical load during each heart-beat. Normal heart load is essential for physiological development and cardiac function. At the same time, excessive load can induce pathologies such as cardiac hypertrophy. While the forces working on the heart as an organ are well-understood, information regarding stretch response at the cellular level is limited. Since cardiac stretch-response depends on the amplitude and pattern of the applied load as well as its timing during the beating cycle, the directionality of load application and its phase relative to action potential generation must be controlled precisely. Here, we design a new experimental setup, which enables high-resolution fluorescence imaging of cultured cardiac cells under cyclic uniaxial mechanical load and electrical stimulation. Cyclic stretch was applied in different phases relative to the electrical stimulus and the effect on cardiac cell beating was monitored. The results show a clear phase-dependent response and provide insight into cardiac response to excessive loading conditions.

## Introduction

Cardiac cells contract against a mechanical load during each heartbeat. The heart performs optimally within a defined range of hemodynamic load and fails if acutely or chronically overloaded (Zimmermann, 2013). Cardiac cells are able to sense their mechanical environment and alter their behavior accordingly, yet, mechanical stimuli are not usually present under standard cell culture conditions. The response of cardiac cells to mechanical loading at the cellular level is a subject of great interest. For the past two decades there has been an effort to study *in vitro* the effect of mechanical loading on cardiac cells and stem cells-derived cardiomyocytes (reviewed in Quinn and Kohl, 2012; Simmons et al., 2012). Sustained stretch (longer than 24 h), either static (Gopalan et al., 2003) or cyclic (Fink et al., 2000; Salameh et al., 2010), was shown to be associated with increased expression level and distribution of gap junction proteins (e.g., Connexin43) and hypertrophy markers (e.g., atrial natriuretic factor, ANF) depending on stretch direction relative to cardiac cell orientation. Several methods were developed to study the response of single cardiac cells to applied load and to elucidate the underlying mechano-chemo-transduction mechanism. These methods include stretching a substrate on top of which cells are attached (reviewed in Quinn and Kohl, 2012), using carbon fibers attached to both ends of a cardiac cell (Le Guennec et al., 1990; Cooper et al., 2000; Prosser et al., 2011) and a “cell-in-a-gel” system whereby isolated cardiomyocytes contract against an elastic three dimensional matrix (Jian et al., 2014). Most of these studies used spontaneously beating cardiac cells with no electrical stimulation. Several recent works combining electrical field stimulation with mechanical stimulation, pointed toward a strong dependence between cardiac cell stretch response and the beating phase when stretch is applied (Nishimura et al., 2006; Morgan and Black, 2014).

Here, we design an experimental setup that allows us to study the effects of applied strain in a controlled direction and at different stages of the cardiac beating cycle. Our experimental setup allows for the application of cyclic mechanical stretch and electrical pulses with a controlled delay between them, while simultaneously monitoring cardiac cell contraction using live confocal microscopy for several hours. Our data demonstrate that after 10–20 min of cyclic mechanical stretch, cardiac cell contraction is shifted with respect to the electrical stimulus with a time shift that depends on the initial timing of load application.

## Results

**Figure 1:**
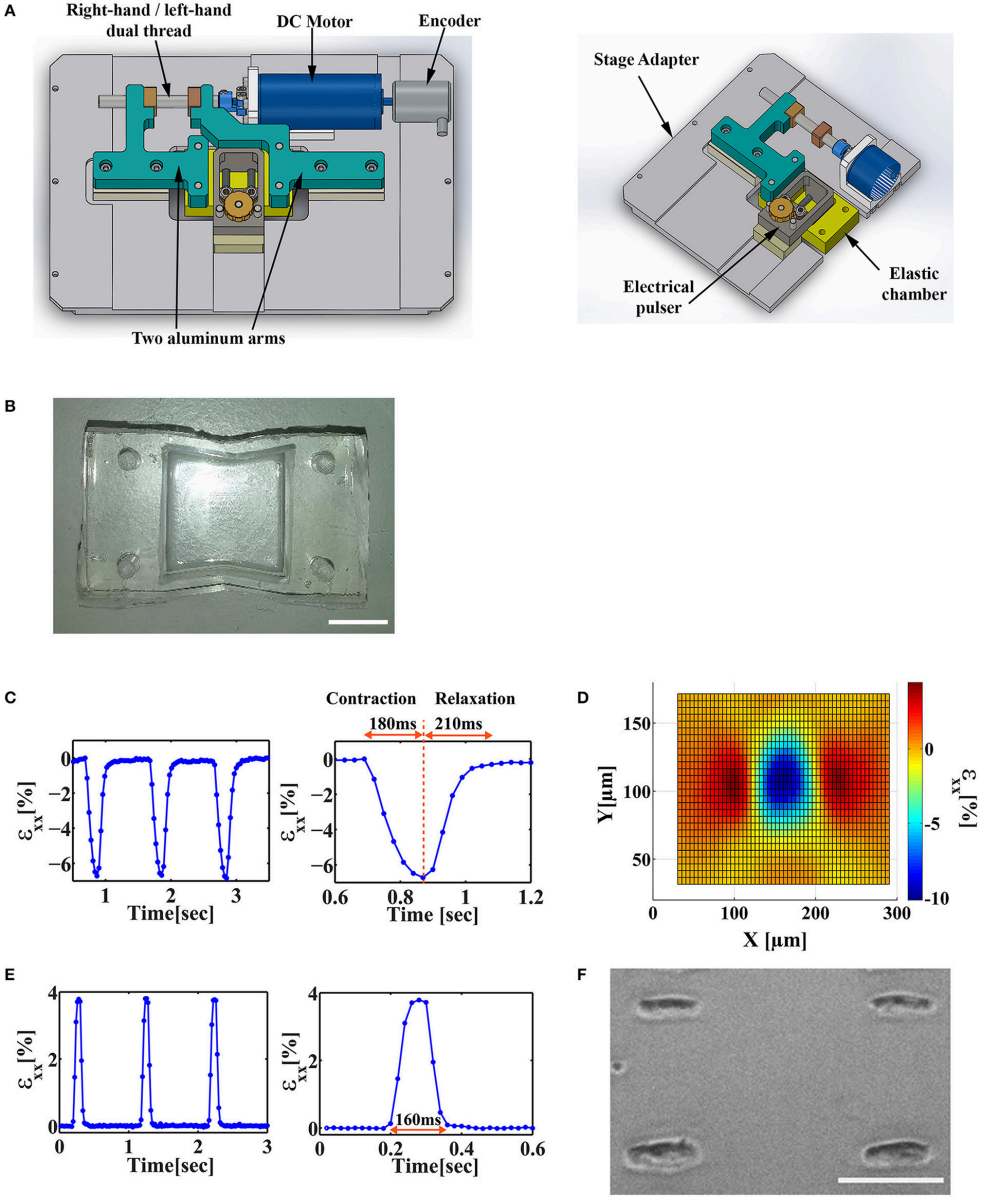
Design of a stretch device that enables the application of stretch at different stages of the beating cycle. **(A)** Solidwork drawings of a microscope mountable stretch device that allows for a synchronized application of uniaxial load and electrical field stimulation. **(B)** An image of a custom-made PDMS “v-shaped” chamber. Scale bar is 1 cm. **(C)** Left*:* The average strain (ε_xx_) generated by a beating cardiac cell along the contraction axis in the underlying substrate. Strain was measured by monitoring the displacement of fluorescent beads embedded in the substrate. Right*:* A single contraction can be divided into a contraction phase (~180 ms) and a relaxation phase (~210 ms). **(D)** The strain field generated by a beating cardiac cell along the contraction axis (x-axis, ε_xx_). **(E)** Left*:* The strain profile generated by the mechanical stretch device as measured by monitoring the displacement of fluorescent beads embedded in the substrate. Right*:* Zoom-in of a single cycle of trapezoid stretch profile with 4% strain amplitude and 160 ms width. **(F)** A representative image of patterned cardiac cells cultured on a PDMS/PA combined chamber. Scale bar is 100 μm.

A widely used method to apply mechanical strain to cells *in vitro* is stretching an elastic membrane to which cells are adhered. We designed a stretch device and used it to apply mechanical stimulation to cells cultured on matrigel-coated polyacrylamide (PA) gel, covalently linked to a flexible Polydimethylsiloxane (PDMS) chamber (Figures 1A,B). Polyacrylamide gel elasticity was tuned to the range of 1–10 kPa. Substrate stiffness in this range was shown to support optimal spontaneous cardiac cell beating for neonatal cardiac cells in culture (Engler et al., 2008; Majkut et al., 2013; Nitsan et al., 2016). By incorporating fluorescent beads in the PA gel and tracking them over time, we could quantify the displacement field generated by the beating cardiac cells (Figures 1C,D) and by the stretch device (Figure 1E). To allow for live cell imaging during cell stretching, the chamber base was made of a thin, 120 μm thickness PDMS film, which is compatible with high fluorescence imaging. The design of the stretch device ensures that the center of the chamber stays within the field of view during stretching. The device is shown schematically in Figure 1A and additional information can be found in the Materials and Methods section.

### Cardiac cells were stretched either in the direction of contraction or in the perpendicular direction

Neonatal cardiomyocytes were cultured on a PDMS/PA combined chamber as described above. We used micropatterning techniques to control cell orientation (Figure 1F) and the direction of stretch application relative to the sarcomere fiber orientation (i.e., contraction axis of the cardiac cell).

**Figure 2:**
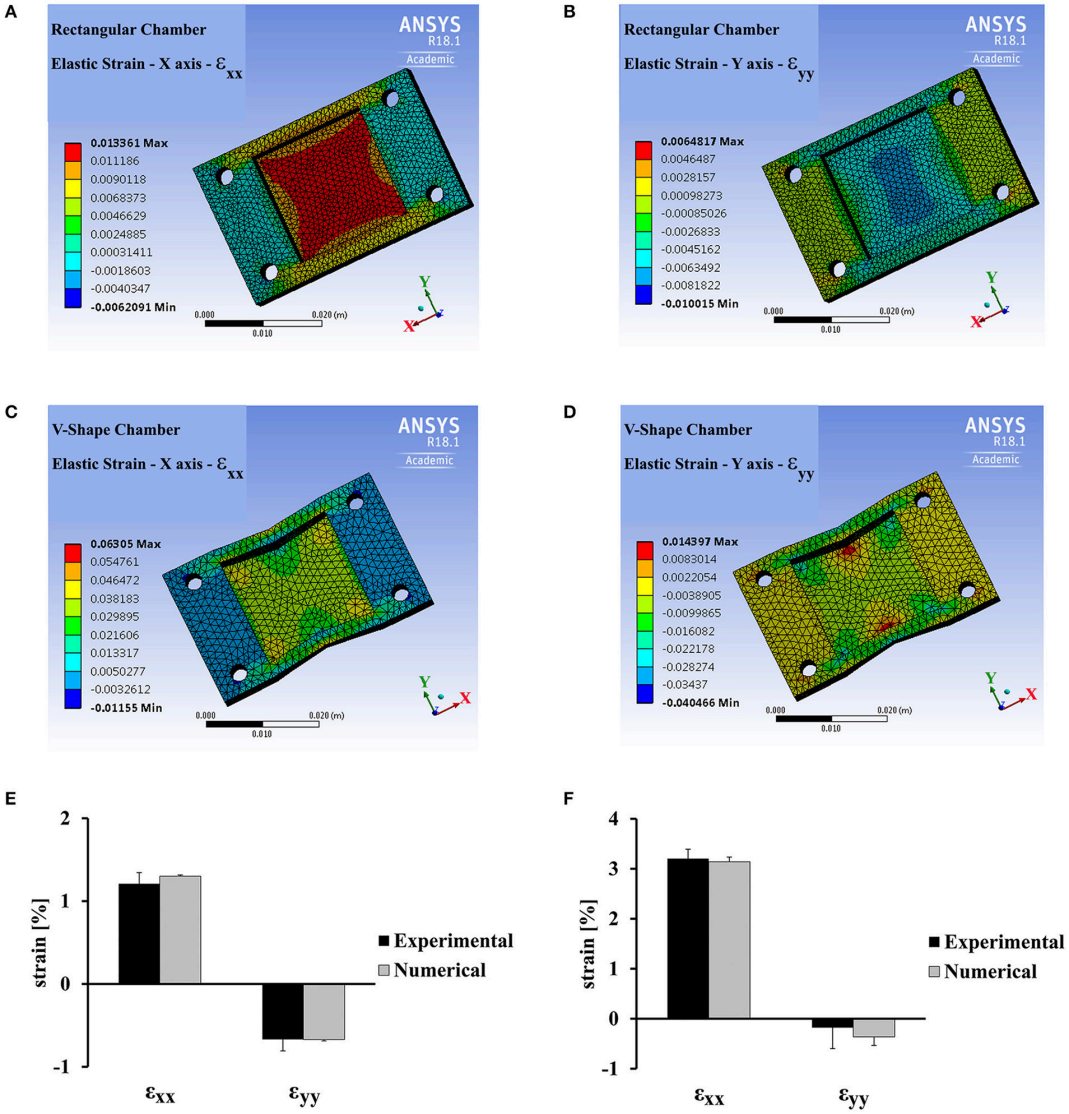
ANSYS analysis and experimental strain field measurements for a rectangular vs. “v-shaped” PDMS chambers. **(A,B)** ANSYS analysis for the strain field generated in a rectangular PDMS chamber along the stretch direction (x-axis, ε_xx_, **A**) and along the perpendicular direction, (y-axis, ε_yy_, **B**) by the application of 1.3% strain using the stretch device. **(C,D)** ANSYS analysis for the strain field generated in a “v-shaped” PDMS chamber along the stretch direction (x-axis, ε_xx_, **C**) and along the perpendicular direction (y-axis, ε_yy_, **D**) by the application of 3% strain using the stretch device. **(E,F)** The average strain generated in a rectangular PDMS chamber **(E)** and a “v-shaped” PDMS chamber **(F)** along the stretch direction (x-axis, ε_xx_) and along the perpendicular direction (y-axis, ε_yy_). Average strain was extracted from ANSYS analysis and by direct experimental measurements. Experimental measurement of strain was done by monitoring the displacement of fluorescent beads embedded in the substrate (for additional details see Materials and Methods). Error bars represent standard deviation. The strain was averaged within a region of 36 mm^2^ in the center of the chamber. This is the same region that was used for cardiac cell measurements in the final experiments.

Since the Poisson ratio of PDMS is 0.49, stretch applied along the x direction, generates strain both in the parallel direction and in the perpendicular direction as shown in Figures 2A,B. The strain generated upon stretching a rectangular PDMS chamber was calculated using ANSYS and measured experimentally by monitoring the displacement of the fluorescent beads embedded in the substrate (Figures 2A,B,E). To have the cells experience strain along a single axis, we designed a “v-shape” chamber. The chamber walls, parallel to the stretch direction, were designed in a “v-shape” with an angle of 11 degrees directed toward the center of the chamber (see Figure 1B). As clearly seen in Figures 2C,D,F, both experimentally and using the ANSYS simulation, the strain along the y-axis (in the perpendicular direction to stretch application) is an order of magnitude smaller than the strain along the x-axis. We can therefore regard this loading process as a nearly-pure uniaxial strain application. In a similar way, we could modify the shape of the chamber to generate other forms of strain application to cells.

### Stretch was applied at different phases during cardiac cell beating cycle

**Figure 3:**
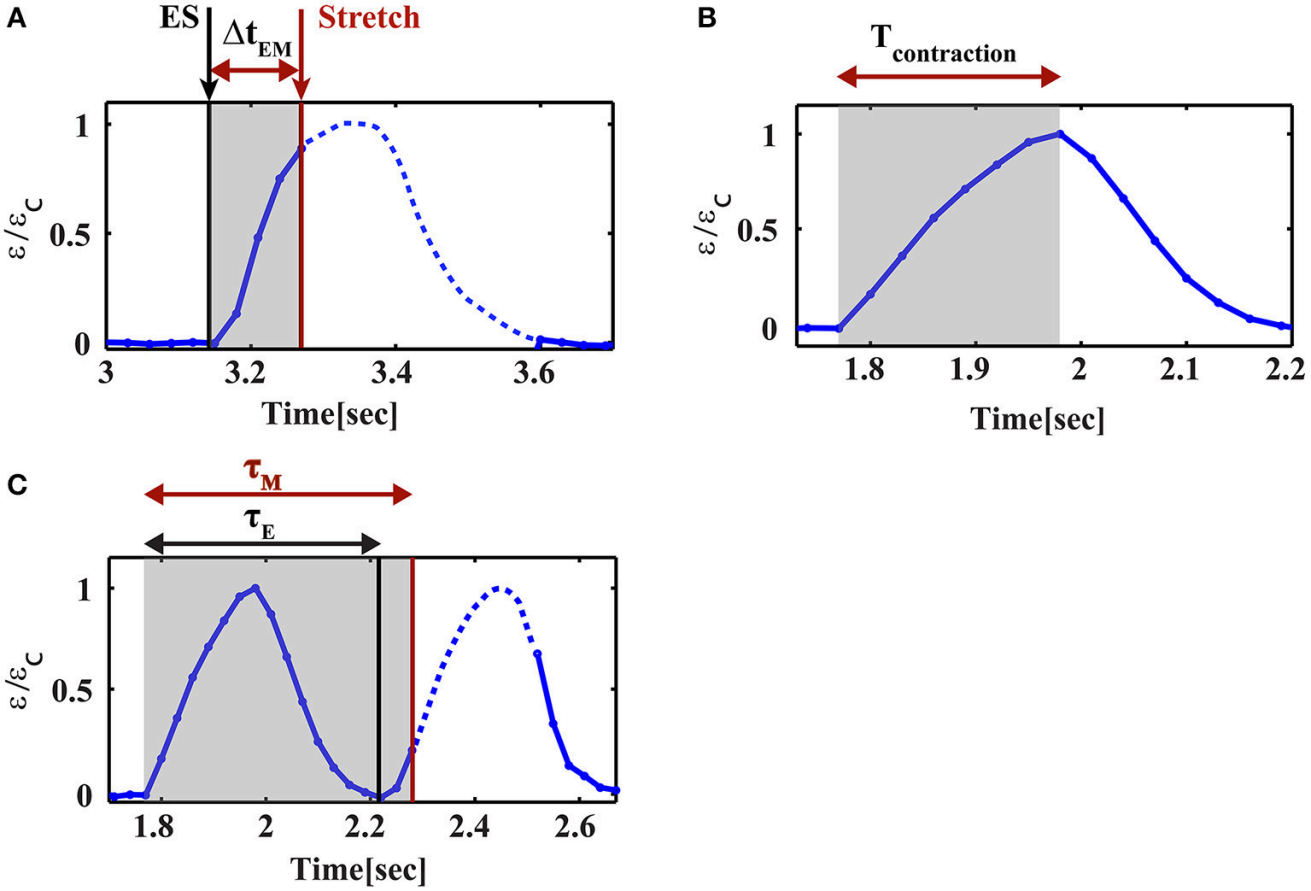
Parameter definitions for cyclic stretch and cell contraction. **(A)** Δt_EM_ is the time delay between the initiation of stretch and the electrical stimulus, **(B)** T_contraction_ is the duration of the contraction phase of the beating cycle, **(C)** τ_M_ is the phase shift of the contraction relative to the mechanical stretch and τ_E_ is the phase shift of the contraction relative to the electrical stimulation. The black lines correspond to the electrical stimulus and the red lines mark the initiation of mechanical stretch.

Cardiac cells were paced using electrical field stimulation at 1 Hz at 25°C. Four percent strain was applied periodically at 1 Hz using a trapezoid profile, 160 ms in width, as shown in Figure 1E. The strain generated by the mechanical device was measured by monitoring the beads embedded in the substrate using a 1x objective (Figures 1E, 2E,F). As shown for a representative cell in Figure 1C, this width is shorter than both the contraction phase (190 ± 9 ms, mean±S.E. for *n* = 26 cells) and the relaxation phase (208 ± 10 ms, mean±S.E. for *n* = 26 cells). The stretch device is computer-controlled to allow us to synchronize mechanical stretching with electrical field stimulation. Using our system, we were able to control the relative time between the electrical field stimulation signal and the initiation of mechanical stretch by the motor. We denote the time between the electrical signal and the mechanical load by Δt_EM_ (Figure 3A). Different values of Δt_EM_ correspond to the application of stretch at different stages of the cardiac cell beating cycle. We denote the length of the contraction phase by T_contraction_ (Figure 3B). For Δt_EM_/T_contraction_ >1, the stretch is applied at the end of the contraction phase and during the relaxation phase, while for Δt_EM_/T_contraction_ <0, the stretch is initiated before cell contraction, during the relaxed state of the cell.

### Cardiac cells were synchronized with the electrical field stimulation in frequency but not in phase when subjected to cyclic mechanical load during the contraction phase

**Figure 4:**
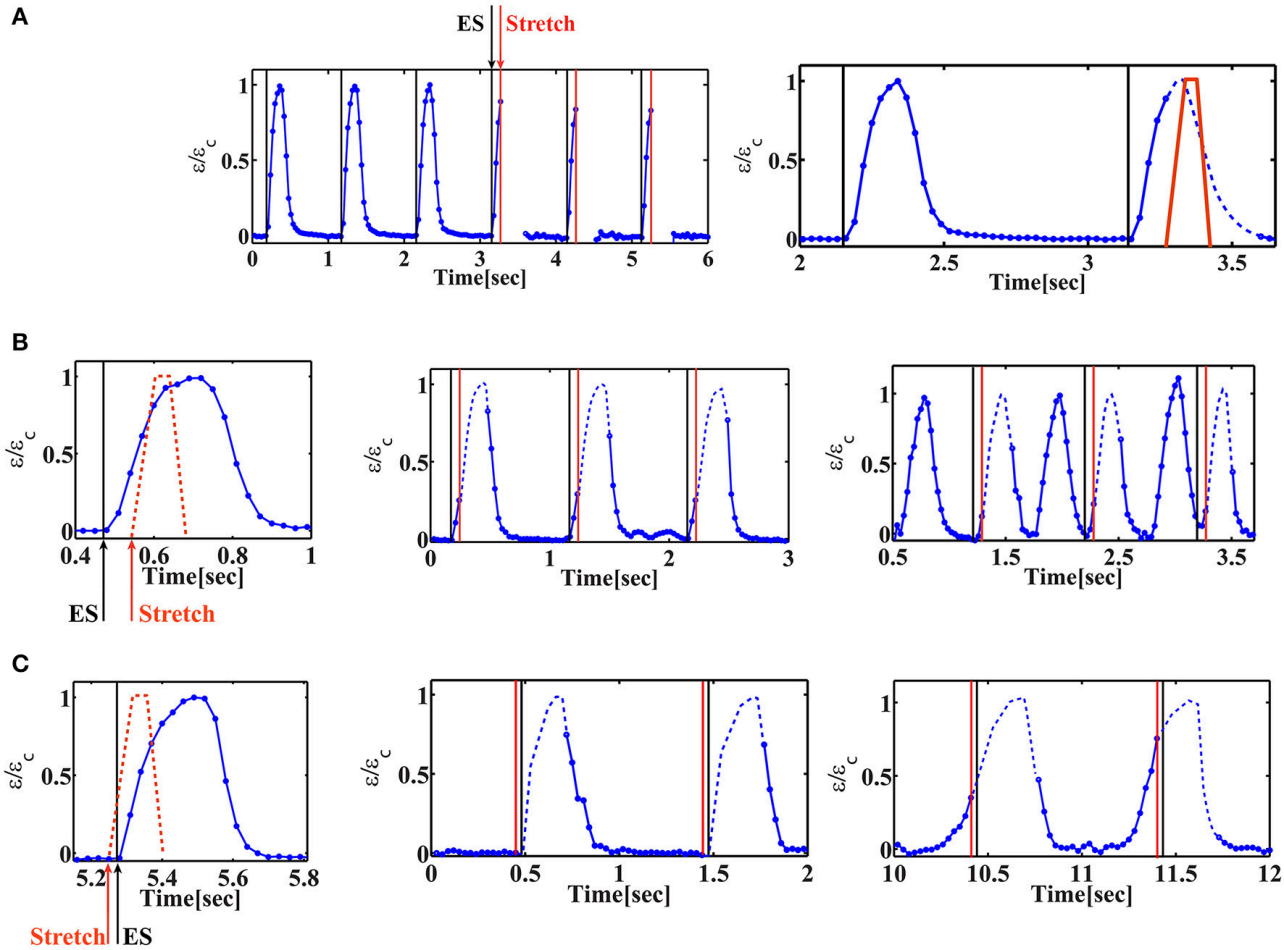
Cardiac cell beating profile under cyclic phase-dependent mechanical loading. Representative beating signals of beating cardiac cells as a function of time for different timings of stretch application. Beating signal was followed by monitoring the displacement of fluorescent beads embedded in the substrate using 40x objective and extracting the strain generated in the substrate. Normalized strain is shown, where ε_c_ is the strain at maximal contraction. When the chamber is stretched, the focus is lost. The signal is recovered when the chamber returns to its original position. The missing parts in the beating signal of the cell are added as a dashed line to guide the eye. **(A)** Left*:* Beating signal of beating cardiac cells before and during cyclic stretch application. The black lines correspond to the electrical stimulus and the red lines mark the initiation of mechanical stretch. As clearly shown, the beating signal of the cell is lost when the chamber is stretched (after three consecutive contractions). Movie S1 shows the displacement of the fluorescent beads embedded in the underlying substrate. Right: the stretch profile is marked (red “trapezoid”) on top of the beating signal and the “missing part” of the beating signal is shown as a dashed line to guide the eye. **(B)** Mechanical stretch is initiated 60 ms after the electrical stimulus (Δt_EM_ /T_contraction_ = 0.27). Left: schematic illustration of the timing of mechanical stretch (shown on top of the beating profile before stretch application). Middle*:* after 1min of continuous cyclic mechanical stretch, no change is observed in the beating profile of the cardiac cell. Movie S2 shows the displacement of the fluorescent beads embedded in the underlying substrate. Right*:* 20 min after continuous cyclic mechanical stretch, the cell contracts 450 ms before the electrical stimulus (τ_E_/T_contraction_ = 1.6). A second contraction is generated following the electrical stimulus. Movie S3 shows the displacement of the fluorescent beads embedded in the underlying substrate. **(C)** Mechanical stretch is initiated 25 ms before the electrical stimulus (Δt_EM_ /T_contraction_ = −0.1). Left: schematic illustration of the timing of mechanical stretch (shown on top of the beating profile before stretch application). Middle*:* after 1min of continuous cyclic mechanical stretch, no change is observed in the beating profile of the cardiac cell. Movie S4 shows the displacement of the fluorescent beads embedded in the underlying substrate. Right*:* 20 min after continuous cyclic mechanical stretch, the cell contracts 240 ms before the electrical stimulus (τ_E_/T_contraction_ = 1.25). Movie S5 shows the displacement of the fluorescent beads embedded in the underlying substrate.

The combined electromechanical signal was applied to the cells for 20 min and both the cells and the fluorescent beads were imaged continuously. In order to accurately monitor the strain generated by the beating cells, we had to increase our resolution by using an 40x objective. Under these conditions, the focus was lost using stretch application and regained at the end of the stretch. This fact enables us to verify the timing of stretch application (Figure 4A and Movie S1). As shown in Figure 4, we were still able to resolve the initiation of cell contraction. Figure 4 demonstrates a representative behavior for two cells under cyclic stretch that was applied during their contraction phase. As clearly shown, after 1 min (60 cycles of mechanical stretch), no change in the beating profile of the cardiac cells was observed (Figures 4B,C and Movies S2, S4). However, after 20 min. of cyclic loading, cells contracted before the electrical stimulus (“early beating”) (Figures 4B,C and Movies S3, S5). In cases where contraction was significantly shifted, the cell was already in the relaxation phase when the electrical stimulus arrived and a second contraction was generated (Figure 4B and Movie S3).

### The phase shift of the contraction depends on the timing and direction of mechanical stretch

**Figure 5:**
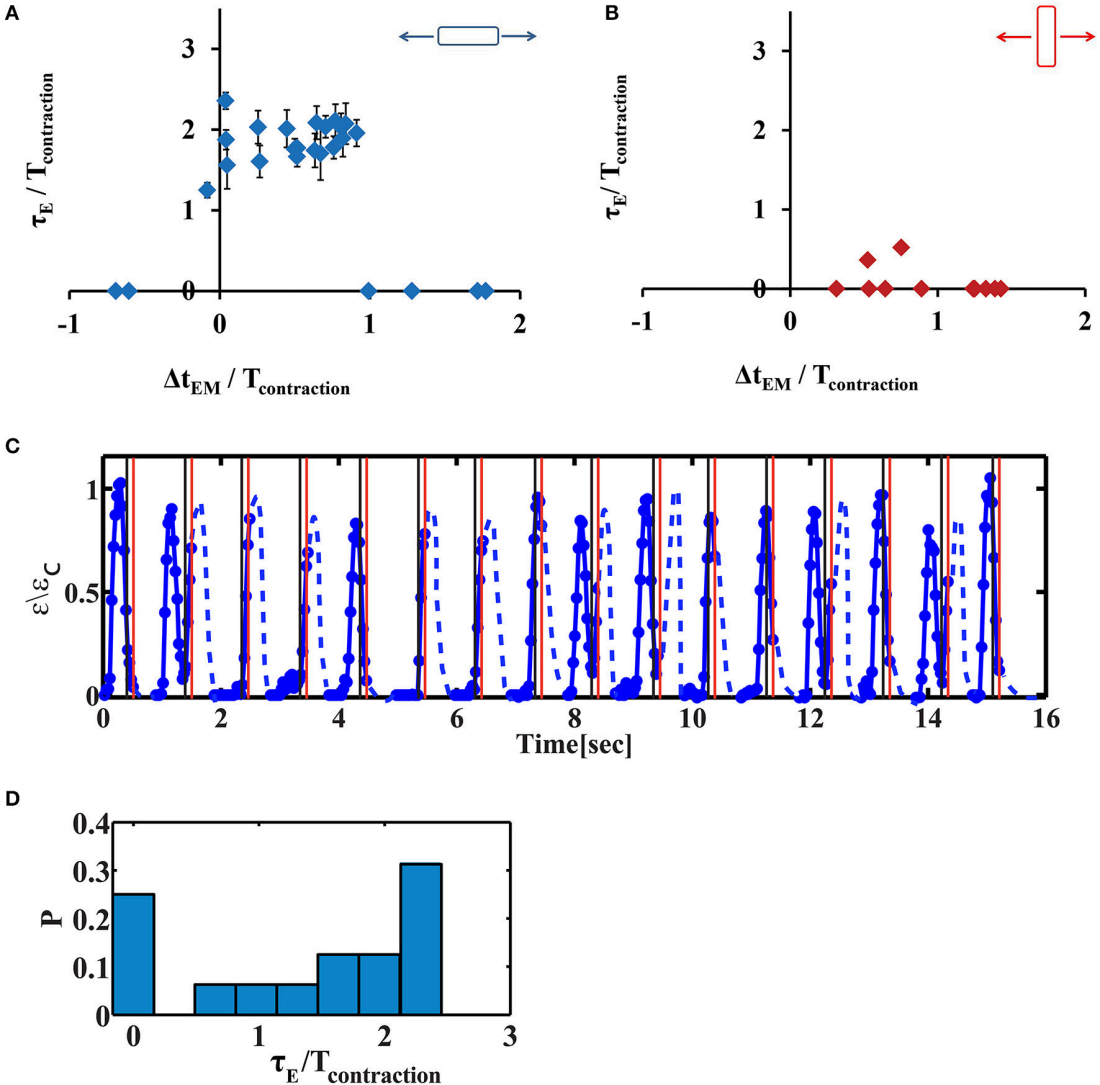
The phase shift of contraction depends on the timing of stretch application. **(A,B)** Average phase shift of cell contraction after 20 min of continuous cyclic loading as a function of the phase of load application. Each point represents a single cell experiment. Stretch was applied either along the direction of cell contraction (**A**, *n* = 26 cells) or along the perpendicular direction (**B**, *n* = 11 cells). The data represents 10 different isolations with at least 3 different replicates for each value of Δt_EM_ from 2–3 different chambers, each from a different isolation. Error bars represent standard error of the contractions that demonstrate a phase shift within a beating signal of a single cell, as shown in **(D)**. Δt_EM_ /T_contraction_ is negative if stretch is applied before the beginning of the contraction phase and larger than one if stretch is applied at the end of the contraction phase and during the relaxation phase. τ_E/_Tcontraction is zero when there is no phase shift. When τ_E/_Tcontraction ~2 indicates that a phase shift on the order of the duration of a full beating cycle occurs, and therefore a second contraction will be generated. **(C)** A representative beating signal of a cardiac cell, 20 min after continuous cyclic stretch which initiates 130 ms after the electrical stimulation, (Δt_EM_ /T_contraction_ = 0.91). Movie S6 shows the displacement of the fluorescent beads embedded in the underlying substrate. **(D)** The histrogram of phase shifts as calculated using the beating signal shown in **(C)**.

We have repeated this experiment with variable time delays between the electrical field stimulation and the mechanical stretch. The results are summarized in Figure 5. We denote the phase shift (the time difference between the initiation of contraction and the electrical stimulus) by τ_E_ (Figure 3C). Similarly, we denote by τ_M_ the time difference between the initiation of contraction and stretch application. “Early beating,” where contraction initiates before the electrical stimulus lead to τ_E_ > 0. τ_E_ = 0 indicates that there is no phase shift, while τ_E/_T_contraction_ ~2 indicates that a phase shift on the order of the duration of a full beating cycle occurs, and therefore a second contraction is generated. As clearly shown in Figure 5A, after 20 min of cyclic stretch applied along the contraction axis, cardiac cells contract before both stimuli (the electrical and the mechanical stimuli). The average phase shift (τ_E_) increased with the overlap of the “trapezoid” stretch with the contraction phase of the beating cell (Figure 5A and Figure S1A). Very differently, when the stretch was applied in the perpendicular direction, no significant influence was found on cardiac cell beating (Figure 5B and Figure S1B). We should note that for a single cell experiment, the beating signal contains a distribution of phase shifts relative to the electrical stimulus, as shown in Figures 5C,D. The distribution is bimodal and contains contractions that are not shifted and contractions that demonstrate a phase shift. The number of contractions that demonstrate a phase shift in the beating signal of cells that were stretched along the contraction axis (during the contraction phase), was higher than 50% (*n* = 20). Differently, when stretch was applied in the perpendicular direction, the number of contractions that demonstrate a phase shift was lower than 7% (*n* = 6).

## Advantages and limitations of the method

The device presented in this work allows for simultaneous application of mechanical and electrical stimulation. Using the combined PDMS/PA chamber, cardiac cell contractile activity can be followed in real time by monitoring fluorescent beads embedded in the substrate. As clearly demonstrated in Figures S2A,B, the strain generated by the beating cardiac cells remains the same after 20 min of electrical field stimulation. In contrast, continuous application of cyclic stretch during the contraction phase (Δt_EM_/T_contraction_ < 1) for 20 min, results in decreased contractile activity (i.e., the strain generated by the cells is reduced) and slower relaxation (Figures S2C–F).

Cardiac fibrosis is characterized by fibroblast accumulation and excessive deposition of ECM proteins that result in increased ventricular stiffness (Travers et al., 2016). The elasticity of a fibrotic tissue is 35–70 kPa, several fold stiffer than the healthy adult heart (~10 kPa) (Engler et al., 2008). The PA substrate rigidity in the PDMS/PA chamber can be easily tuned and matched to the stiffness of a fibrotic tissue or to the elasticity of the heart in different developmental stages (Majkut et al., 2013).

The shape of the chamber was chosen to ensure that the cell experiences strain only along a single axis. Nevertheless, other forms of stretch can be generated by modifying the shape of the chamber. For example, using an angle of 20 degrees (instead of 11), the cell will experience extensive strain along both the x and y axes (ε_xx_ = 2.5% ε_yy_ = 4.5% respectively). The patterning protocol described, allows to accurately control the direction of stretch application relative to the direction of the cell contraction axis.

The ability to perform high resolution fluorescence microscopy in real time, can be used to measure calcium transients as shown in Figure S3 and Movies S7, S8. However, since the focus is lost during the stretch in high magnification (40x), only part of the calcium signal can be captured during the stretch (Figure S3B). In order to capture the full calcium signal, one can either use lower magnification or pause the stretch device during image acquisition.

The right-hand/left hand dual thread pitch used in the design is 0.8 mm and therefore one full rotation of the motor generate 1.6 mm axial displacement between the arms and 1.6/20 mm = 0.08 = 8% strain for the chamber we used (20 mm in length). This theoretical estimation was verified by direct measurement of the displacement field of fluorescent beads that were embedded in the PDMS/PA chamber.

The experiments presented in this work were conducted under combined electrical and mechanical stimulation of 1 Hz. However, higher frequencies can be applied (Figure S4). The maximal motor velocity is 6,000 RPM (~0.8%/ms), the maximal acceleration/deceleration rates are: 6,000 RPM/ms (0.8%/ms^2^) and 300 RPM/ms (0.04%/ms^2^) respectively and the time required for the motor to change direction is 40 ms. Using these parameters the maximal frequency of device operation can be estimated for a specified amount of strain. For example, for 20% strain, the motor will reach its target speed of 6,000 RPM in 1 ms and during that time the chamber will be stretched by 0.4%. Next, the chamber will be stretched with the target velocity up to 12% strain and then for 20 ms the motor slows down and the chamber is stretched up to 20%. Same timing is required for the relaxation. Additionally, in the current configuration, a change in motor direction requires 40 ms (this time can be reduced to 1 ms if real-time software will be used), which leads to a maximal frequency of 6 Hz for 20% strain. For 4% strain (as used in this work), the maximal frequency is 9 Hz.

## Concluding remarks

Cardiac cells are subjected to mechanical load during each heart-beat and excessive loading can lead to pathologies. While the forces working on the heart as an organ are well understood, the response of cardiac cells to mechanical forces on the cellular level is still not completely understood. The platform we setup, allows us to study the response of cardiac cells to mechanical forces applied at different stages of the cardiac beating cycle. It allows monitoring of cardiac cell beating in real time, using high resolution optical microscopy, while applying cyclic mechanical loading under electrical field stimulation, in a controlled direction and timing. Our data demonstrate that after 10–20 min of cyclic mechanical stretch, applied along the cell contraction axis, cardiac cells contract with a phase shift relative to the electrical stimulus. The phase shift is the largest if the load is applied during contraction and decreased dramatically if load is applied during the relaxation phase. The fact that this contraction initiates before the electrical stimulus and that it takes 10–20 min to obtain this phenomena, may suggest that cyclic stretch induces alterations in the biochemical machinery that governs spontaneous cardiac cell beating. The observation that within a single beating signal, we obtain a distribution of phase shifts, further supports the hypothesis that the cyclic loading interfere with spontaneous beating which is intrinsically stochastic. Recent observations demonstrate increased frequency of calcium sparks as a result of mechanical stretch (Iribe et al., 2009; Prosser et al., 2011). An increase in spark frequency may be an indication for a change in the kinetics of the channels within the “calcium clock” machinery. Future experiments are required to demonstrate this concept. These include following calcium sparks and applying stretch in the presence of inhibitors for proteins that were suggested to play a key role in cardiac cell's mechanotransduction (Jian et al., 2014; Prosser and Ward, 2014). Our setup enable monitoring changes induced in the contractile activity of cells (Figure S2) and in their sarcomeric cytoskeleton (Figure S5 and Movie S9) as a result of cyclic stretch and therefore can be used in the future as an *in-vitro* model to study development of pathologies where the heart experiences excessive load during specific phases of the cardiac beating cycle. For example, pressure overload which is associated with excessive load mainly during systole, results in sarcomere addition in parallel with a relative increase in the width of individual cardiomyocytes (concentric hypertrophy). In contrast, volume overload which results in excessive load mainly during diastole, leads to addition of new sarcomeres in series with an increase in the length of the cells (eccentric hypertrophy) (Mutlak and Kehat, 2015). Using the presented setup, both of these scenarios can be mimicked and the addition of new sarcomere units can be followed.

## Materials and methods

### Animal use

All laboratory procedures conform to the Guide for the Care and Use of Laboratory Animals published by the U.S. National Institutes of Health. Animal usage was approved by the Animal Care and Use Committee of the Technion, Israel institute of Technology.

### Stretch device

We designed a custom-built stretching device, which transfers torque generated by a DC motor using a right-hand/left-hand dual thread into a linear motion of two aluminum arms attached to an elastic silicon chamber. The arms are mounted to frictionless tables that constrain their movement along a single axis (Figure 1A). The symmetrical motion of the arms ensures that the central part of the elastic chamber stays within the imaging field of view during the stretching process. The device is mounted to a microscope stage adapter which fits to an inverted spinning disc confocal microscope stage (see below). For electrical stimulation, two carbon electrodes (Graphite 3.05 mm; Alfa Aesar) were placed 1 cm apart and held by a frame made of Delrin located just above the chamber. The position of the electrodes relative to the bottom of the chamber is adjustable. The dimensions of the chamber are 20 × 20 mm and the thread pitch is 0.8 mm. Production drawings will be provided upon request.

The motor used was EC-4pole 22 (P/N 323220; MAXON MOTORS) with an encoder, SCH16F 2000CPT (P/N 461212; MAXON MOTORS), a controller EPOS 70/10 (MAXON MOTORS) and a 1,000 W power supply, RSP-1000-48 (MEAN WELL).

Function generator model Stanford DS345, with a frequency range of 1 μHz to 30.2 MHz and frequency resolution of 1 μHz, was used to synchronize the operation of the motor controller and the electrical pulser. Electrical field stimulation unit model SIU-102 (warner instruments) was used to generate electrical field stimulation pulses. Electrical pulse amplitude was 80 mA with 10 ms duration. We used a custom-built labview interface to control the delay between the trigger for the mechanical stretching device and the trigger to the electrical field stimulation unit. A custom-built labview interface allows us to control the following parameters for stretch and release profile: Initial and final positions of the motor, acceleration rate (RPM/s), velocity (RPM), Deceleration rate (RPM/s), and pausing time between stretch and release (ms). The values used for the acceleration rate, velocity, deceleration rate, and pausing time are: 100,000 RPM/s, 3,000 RPM, 100,000 RPM/s, and 5 ms, respectively.

### PDMS chamber preparation

PDMS chamber walls were prepared by thoroughly mixing a 10:1 ratio of Dow Corning Sylgard 184 silicone elastomer and curing agent resulting in a Young's modulus of E~1MPa (Johnston et al., 2014). The mixture was cast into a 3D printed mold (made of clear resin GPCL04, FORMlabs, see also Figure S6 in the Supplementary information) and cured at 80°C for 2 h. Next, 10 g of PDMS solution were spin-coated on a silicon wafer at 460 RPM for 40 s to achieve thickness of 120 μm. Immediately after, the walls were placed on top of the 120 μm membrane and the full structure was cured at 80°C for 75 min.

### Cardiac cell micropatterning

#### PDMS stamp: microfabrication and soft lithography

The patterns were designed using CleWin 5 (PhoeniX Software, NL). Two rectangle sizes were used: either 20 × 50 μm or 20 × 100 μm. Each rectangle represents a place that will be occupied by a cardiac cell. Chrome masks were manufactured by Delta Mask (Delta Masks BV, NL). A Si master with geometric patterns was fabricated using standard photolithographic technique as previously described (Tang et al., 2012). PDMS prepolymer was obtained via mixing Silicone elastomer and curing agent at 10:1 ratio (Dow Corning, Midland, MI) and degassing the mixture in a desiccator for 30 min. The prepolymer solution was poured over the Si master mold and cured in the oven at 80°C for 2 h. The elastomeric stamp was then peeled off carefully and cut in 1 × 1 cm^2^ squares for micropatterning.

#### Intermediate patterned glass coverslips

Matrigel (Corning Inc, NY) was diluted 1:10 in L15 medium (Thermo Fisher). One hundred and sixty microliters of the solution was used to cover the surface of the elastomeric microstamps and incubated for 1 h at room temperature. Following incubation, the solution was aspirated, and the surface of the stamps was dried with a low stream of N_2_ gas. The stamps were then used to micropattern clean glass coverslips, via microcontact printing. The stamp was brought into complete conformal contact with a 16 mm round glass coverslip substrate for 5 min at room temperature. Small weights (26 g) were placed over the PDMS stamp to aid complete protein pattern transfer from the PDMS stamp to the glass (Tang et al., 2012).

#### Activation of the PDMS chamber

PDMS membrane (chamber base) were then impregnated with the UV-reactive molecule benzophenone as previously reported (Simmons et al., 2013). Briefly, a solution of 10 wt%/vol benzophenone (99% pure, Acros Organics, NJ) dissolved in a water/acetone mixture (35:65 w/w) was placed on the silicone membrane for 120 sec. The silicone membrane were immediately rinsed with methanol for 3 times and dried with nitrogen.

#### Patterning polyacrylamide -PDMS chambers

Polyacrylamide (PA) gel was prepared by adopting procedures outlined in (Pelham and Wang, 1997; Dembo and Wang, 1999; Sabass et al., 2008). Briefly, Polyacrylamide/Bis-acrylamide 7.5/0.03% solution was mixed with 0.05% Ammonium Persulfate and 0.4% Temed (Bio-Rad, CA). Fluorescent beads were added to allow for mechanical deformation analysis (0.02% of 0.2 μm carboxylated dark red fluospheres (Life Technologies) or 0.004% 4 μm sulfate red fluospheres (Life Technologies) for imaging at 40x magnification or 10x magnification respectively). Six microliters of mixed PA solution was applied to the center of the PDMS chamber and covered with, matrigel patterned glass coverslip, to form a thin film. The patterned matrigel on the coverslips was transferred to the surface of the polyacrylamide substrates during polymerization. The chamber was then exposed to ultraviolet light (λ = 365 nm) for 135 s. Films were left to polymerize for 6 additional minutes in room temperature. Following polymerization, the PDMS-PA chamber was incubated with phosphate buffered saline (PBS, pH 7.4) for 60 min and the top coverslips were carefully removed with a razor blade (Ribeiro et al., 2015).

### Cell culture

Neonatal cardiomyocytes were isolated from 0-day-old Sprague Dawley (SD) rat pups using the Neonatal Cardiomyocyte Isolation System (Worthington, NJ) according to the manufacturer's instructions as described before(Nitsan et al., 2016). Briefly, hearts were rinsed with ice-cold HBSS and trypsinized for 16 h at 4°C. Following trypsinization, hearts were incubated with trypsin inhibitor at 37°C, and then with collagenase solution for 30 min. Cells were triturated, pelleted and resuspended in L15 media (Worthington, NJ). Cells were then subjected to Percoll gradient for separation of cardiomyocytes from non-myocytes. Isolated cardiomyocytes were resuspended with culture media: F10 media (Sigma-Aaldrich, MO) supplemented with 5% fetal calf serum (Biological-Industries, Israel), 5% donor horse serum (Life-technologies, CA), penicillin 10 μ/ml, streptomycin 0.1 mg/ml, 5-Bromo-2′-deoxyuridine (5-BrdU) 0.05 mg/ml, and CaCL_2_ 1 mM (Signa-Aldrich, MO). Approximately 6 × 10^5^ cells were plated on matrigel patterned PDMS/PA combined chamber. Cardiomyocytes were cultured for 4–5 days.

### Visualization calcium transients in cultured myocytes

TroponinT-GCaMP5-Zeo (Addis et al., 2013) was a gift from John Gearhart (Addgene plasmid # 46027). GCaMP5 open reading frame was amplified using forward primer containing EcoRI site and reverse primer containing NotI site: Fr primer ACGGAGAATTC GCT ATG GGT TCT CAT CAT CAT C, Rv primer TCCTTTTG CGG CCG CTA TCA GTC CTG TTC CTC AGC. GCaMP5 PCR was cloned into entry vector pENTR3C using EcoRI and NotI restriction enzymes to create pENTR3C-GCamp5. LR recombination was performed using entry vector pENTR3C-GCaMP5 and destination vector pAd/CMV/V5-DEST (ThermoFisher Scientific) to yield pAd-GCaMP5. The pAd plasmid pAd-GCaMP5 was linearized by digestion with PacI and transfected using PolyJet (SignaGen Laboratories) into HEK293A cells. The resultant adenovirus was further amplified by infection of HEK293A cells and was purified by ViraBind Adenovirus Miniprep Kit (Cell Biolabs, Inc.)

Twenty-four hours following plating, cells were transduced with 100 MOI, calcium transients were measured following 48 h of transduction.

### Visualization of F-actin in cultured myocytes

Twenty-four hours following plating cells were transduced with 100 MOI rAV ^CMV^-LifeAct-TagRFP (ibidi, Germany).

### Spinning disc confocal microscopy

Imaging was done using a Nikon Ti-E inverted microscope equipped with two Evolve EMCCD cameras (Photomatrix), 4 laser lines (405, 491, 561, 642), phase contrast and Yokagawa spinning disc confocal. The dish was maintained at 25°C with 5% CO_2_/95% air using a cage incubator (Okolab). Imaging was done using 40x 0.95 NA (air) PlanApo Lambda objective (Nikon). Fluorospheres were excited using 642 nm laser (Vortran), Lifeact-RFP was excited with a 561 nm later (Cobolt) and GCaMP5 was excited with a 491 nm laser (Cobolt). Simultaneous imaging of the 491 and 642 nm channels was done using Photometrics DV2 system. Stream acquisition (using Metamorph software) was performed before the cyclic loading and during 20 min of continuous cyclic loading.

### Data analysis

Basic image analysis is done using ImageJ and custom-writen matlab codes. Mechanical deformations were analyzed using a Digital image processing (DIC) code (Jones, 2013).

### Finite element analysis

The PDMS chambers were analyzed using commercial software (ANSYS workbench R18.1). The “rectangular” and “V-shape” chambers were discretized into 37,634 and 18,335 elements respectively. The PDMS was modeled as a linear elastic and isotropic material with Poisson ratio of 0.49 and Young's modulus of 1 MPa. The boundary condition in this study was that both chamber ears mounted rigidly by their mounting holes (simulating rigid attachment to metal pins at the experiment setup) and X displacement was applied symmetrically to stretch the platform. The resulted normal strain at X and Y axis captured and the membrane mean strain of each axis was calculated at the zone of interest at the center of the membrane (36 mm^2^).

## Author contributions

ST designed research and wrote the manuscript. LD and SD performed experiments. LD analyzed the data. ST supervised the research. All authors discussed and help prepare the manuscript.

### Conflict of interest statement

The authors declare that the research was conducted in the absence of any commercial or financial relationships that could be construed as a potential conflict of interest.
