# Supplementary material for: The Influence of the Timing of Cyclic Load Application on Cardiac Cell Contraction
Source: Front Physiol. 2018 Jul 18;9:917. doi: 10.3389/fphys.2018.00917 (PMC6058596; doi:10.3389/fphys.2018.00917)
Supplement: Supplementary file 10 [file Data_Sheet_1.PDF]

Supplementary Material

The influence of the timing of cyclic load application on cardiac cell contraction

Lior Debbi[1], Stavit Drori[1] and Shelly Tzlil[1*]

[1]Faculty of Mechanical Engineering, Technion – Israel Institute of Technology, Haifa 32000 Israel.

* To whom correspondence should be addressed: Email: shellytz@technion.ac.il

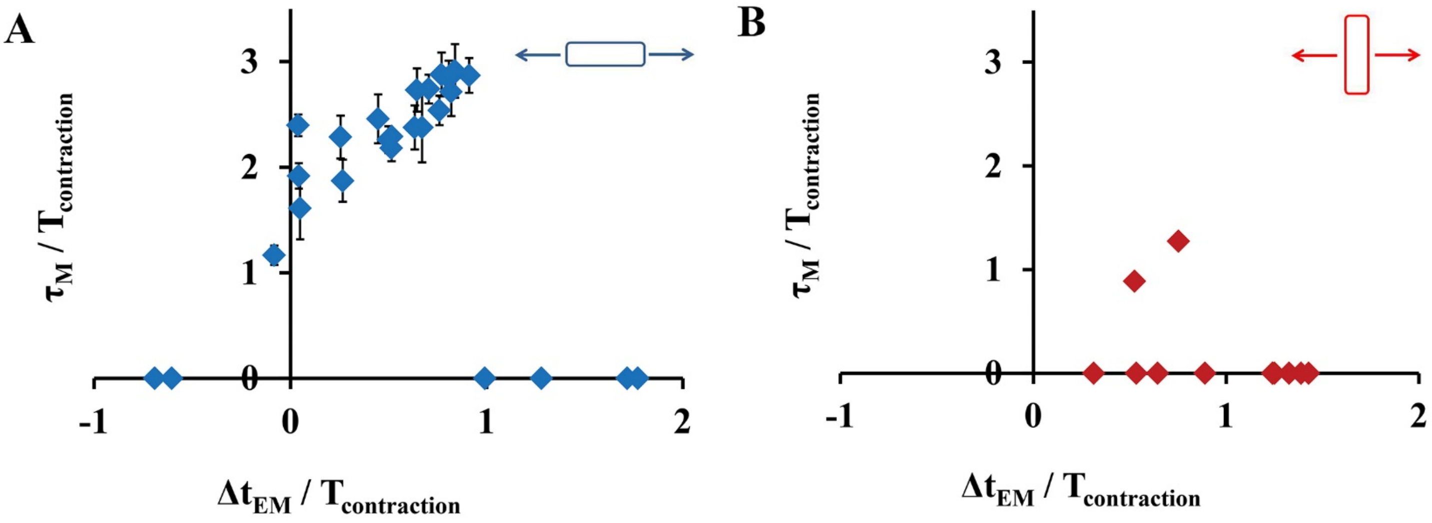

Figure S1: The phase shift of contraction depends on the timing of stretch application. Average phase shift of cell contraction after 20 min of continuous cyclic loading as a function of the phase of load application. Each point represents a single cell experiment. Stretch was applied either along the direction of cell contraction (**A**, n=26 cells) or along the perpendicular direction (**B**, n=11 cells). Parameters are defined in Fig. 3. Δt_{EM} /$T_{contraction}$ is negative if stretch is applied before the beginning of the contraction phase and larger than one if stretch is applied at the end of the contraction phase, during the relaxation phase. τ_{M}/$T_{contraction}$ is zero when there is no phase shift.

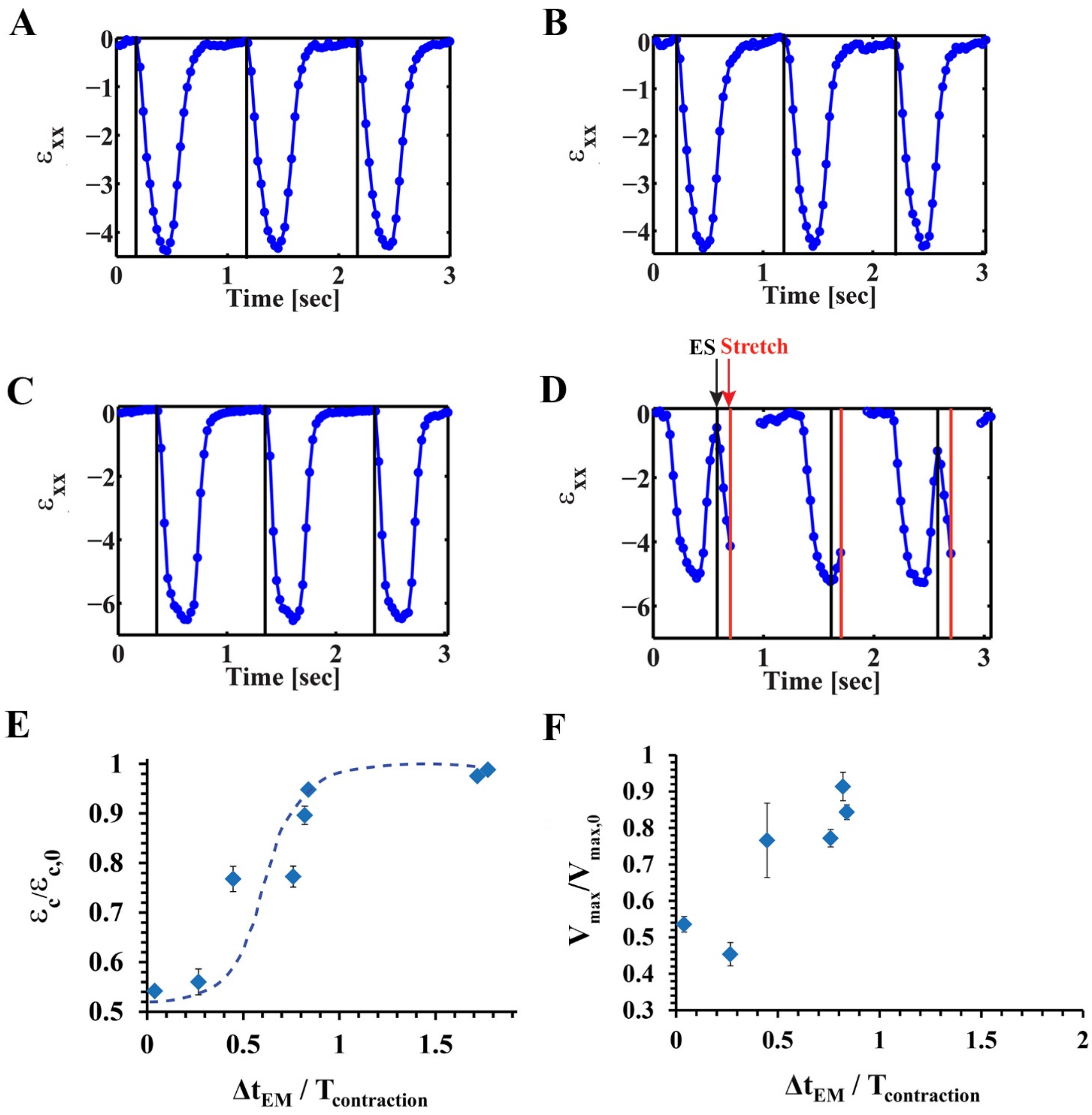

Figure S2: Cardiac cell contractile activity depends on the timing of stretch application. (A-B): A representative signal for the average strain generated by a beating cardiac cell along the contraction axis (ε_{xx}) as a function of time, after 15sec of electrical field stimulation (**A**, <ε_c >= -4.3%±0.01) and after 20min. of electrical field stimulation (**B**, <ε_c>= -4.3%±0.015). ε_c is the strain at maximal contraction. **(C-D)**: A representative signal for the average strain generated by a beating cardiac cell

along the contraction axis (ε_{xx}), in an experiment where mechanical stretch was initiated along the direction of cell contraction 120msec after the electrical stimulus (Δt_{EM} /$T_{contraction}$=0.759). Signal are shown before the application of mechanical stretch (**C**, $<\varepsilon_c>$= -6.4%±0.02) and after 20min. of continuous cyclic mechanical stretch (**D**, $<\varepsilon_c>$= -4.9%±0.13). The black lines correspond to the electrical stimulus and the red lines mark the initiation of mechanical stretch. **E:** Average strain generated by a beating cardiac cell along the contraction axis after 20 min of continuous cyclic loading (ε_c), normalized by the strain generated by the cell before load application ($\varepsilon_{c,0}$) as a function of the phase of load application. Stretch was applied along the direction of cell contraction. Each point represents a single cell experiment (n=8 cells). Error bars represent standard error. **F:** Average maximum relaxation speed of a beating cardiac cell after 20 min of continuous cyclic loading (V_{max}), normalized by the average maximum relaxation speed before load application ($V_{max,0}$) as a function of the phase of load application. When the chamber is stretched, the focus is lost and the signal is recovered when the chamber returns to its original position. Therefore, the relaxation profile could be followed only for contractions where the phase shift is significant ($\tau_M/T_{contraction}$ >1.5). Each point represents a single cell experiment (n=6 cells). Error bars represent standard error.

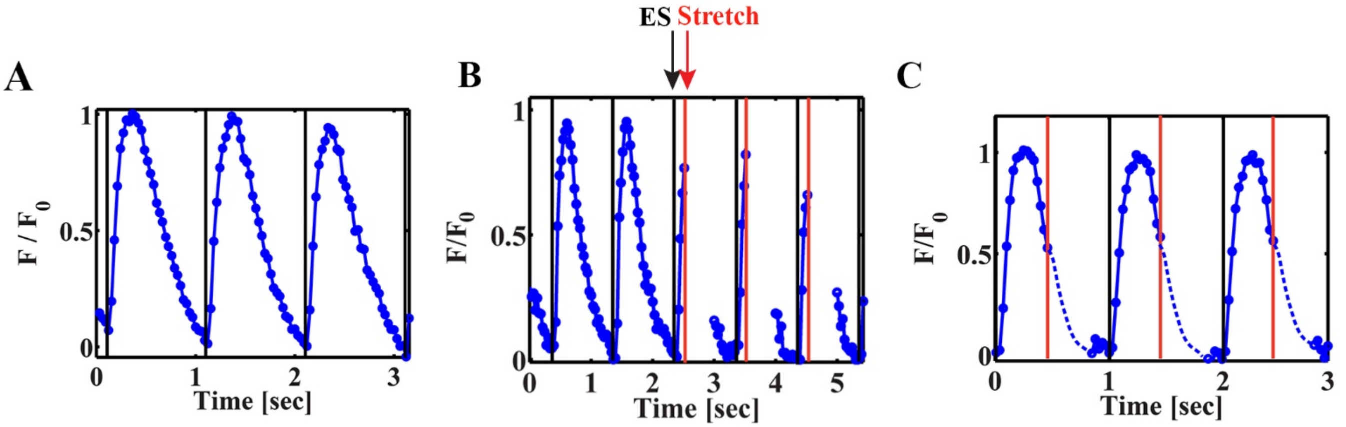

Figure S3: Cardiac cell calcium transients: Calcium transient measurement for a beating cardiac cell transduced with rAV^{CMV}-GCaMP5, a genetically encoded calcium indicator. The black lines correspond to the electrical stimulus and the red lines mark the initiation of mechanical stretch. When the chamber is stretched, the focus is lost. The signal is recovered when the chamber returns to its original position. **A:** Normalized calcium signals (F/F_0) before the activation of cyclic mechanical stretch, the full calcium transient can be observed (Movie S7). **B:** Normalized calcium signals (F/F_0) of a beating cell before and during cyclic stretch application. Stretch was initiated at t=2.52sec, 120msec after the electrical stimulus ($\Delta t_{EM} / T_{contraction}$=0.62), the signal is lost when the chamber is stretched and only part of the relaxation of the calcium signal can be observed (Movie S8). **C:** Normalized calcium signals (F/F_0) after 1min. of cyclic stretch that was initiated at t=0.51sec, 450msec after the electrical stimulus ($\Delta t_{EM} / T_{contraction}$= 1.875). The calcium upstroke, transient peak and the start of the relaxation can be clearly observed. The missing parts in the calcium signal of the cell are added as a dashed line to guide the eye.

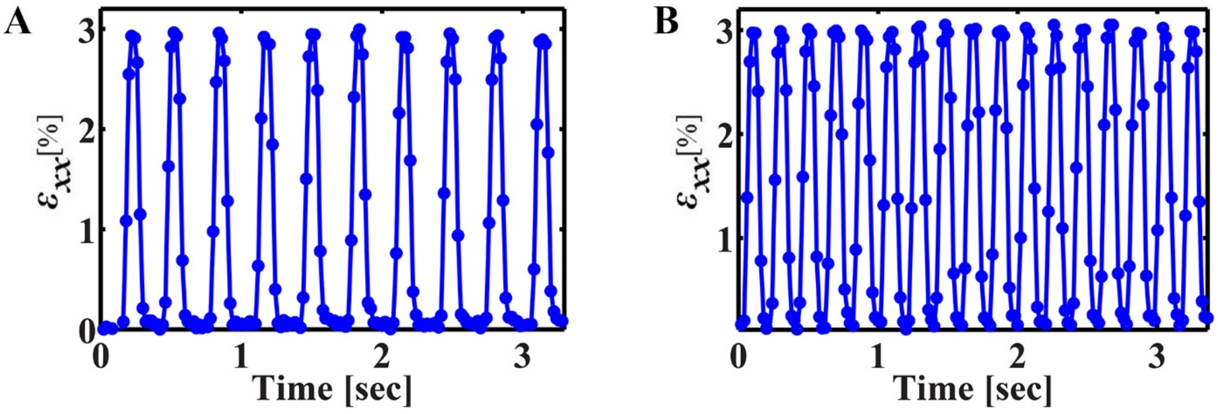

Figure S4: Device operation in different frequencies. Strain profile generated by the mechanical stretch device as measured by monitoring the displacement of fluorescent beads embedded in the substrate at 3Hz (**A**) and 5Hz (**B**).

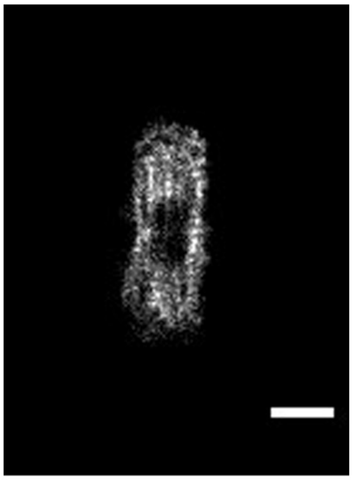

Figure S5: Live beating of actin-labeled cardiomyocytes. Cardiomyocytes were transduced with rAV^{CMV}-LifeAct-RFP to allow for live imaging of sarcomere structure (see also Movie S9). Scale bar is 10μm.

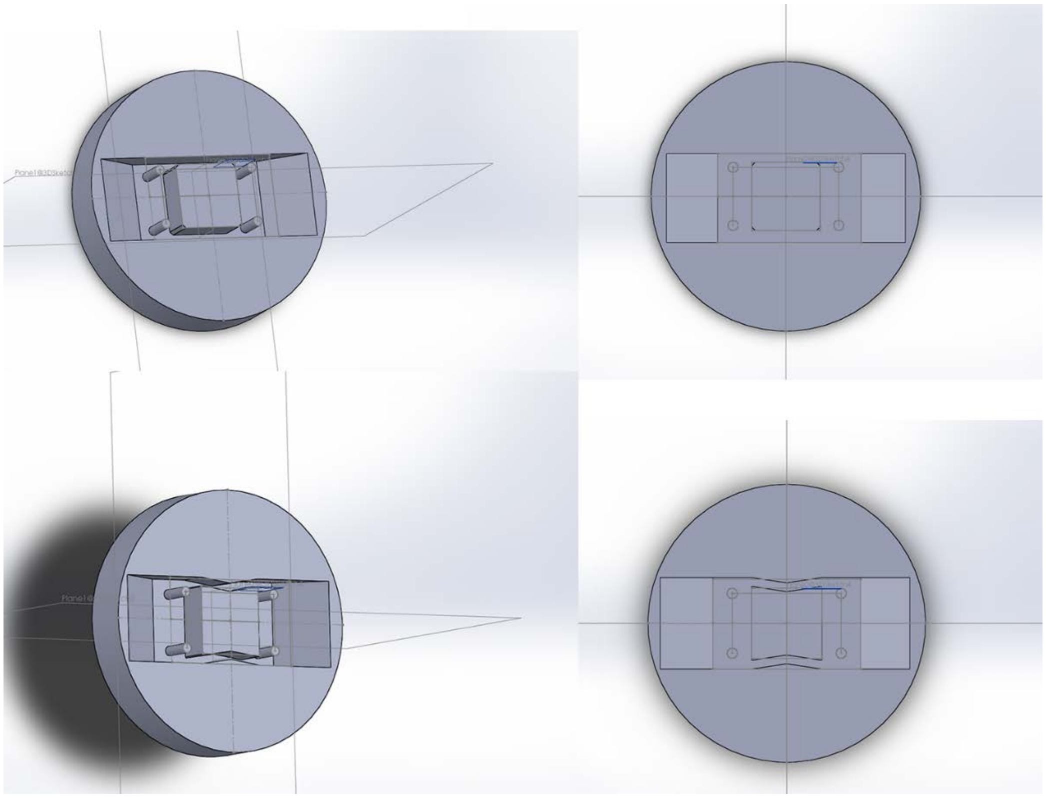

Figure S6: PDMS chamber molds. Chambers were modelled using solidworks 3D CAD software (top: rectangular chamber mold, bottom: v-shaped chamber mold). Molds were printed using a Form2 3D printer (FORMlabs) with clear GPCL04 resin. Printer files are provided in STL format.

MOVIE LEGENDS

Movie S1: Bead displacements generated by beating cardiac cells paced electrically before and during application of mechanical stretch Time lapse imaging of the deformation field generated by beating cardiac cells on a flexible substrate. Mechanical deformations are measured by monitoring the position of 0.2μm fluorescent beads embedded within the gel. The corresponding graph for the normalized strain generated by the beating cells as a function of time is shown in Figure 4A. The cells are electrically paced, mechanical stretch is initiated at t=3.27sec, 130msec after the electrical stimulus (Δt_{EM} /$T_{contraction}$=0.72). Notice that the fluorescence signal of the cell is lost when the chamber is stretched. The fluorescent signal is recovered after the chamber returns to its original position (at the end of the 'trapezoid' stretch). The movie is played in real time. Scale bar is 25μm.

Movie S2: Bead displacements generated by beating cardiac cells after 1 minute of cyclic stretch applied 60msec after the electrical stimulus (Δt_{EM} /$T_{contraction}$=0.27) Time lapse imaging of the deformation field generated by a cardiac cell on a flexible substrate. Mechanical deformations are measured by monitoring the position of 0.2μm fluorescent beads embedded within the gel. The corresponding graph for the normalized strain generated by the beating cells as a function of time is shown in Figure 4B-*middle*. The cells are electrically paced, mechanical stretch is initiated at t=0.21sec, 60msec after the electrical stimulus (Δt_{EM} /$T_{contraction}$=0.27). No change is observed in the beating profile of the cardiac cell. The movie is played in real time. Scale bar is 25μm.

Movie S3: Bead displacements generated by beating cardiac cells after 20 minute of cyclic stretch applied 60msec after the electrical stimulus (Δt_{EM} /$T_{contraction}$=0.27) Time lapse imaging of the deformation field generated by a cardiac cell on a flexible substrate. Mechanical deformations are measured by monitoring the position of 0.2μm fluorescent beads embedded within the gel. The

corresponding graph for the normalized strain generated by the beating cells as a function of time is shown in Figure 4B-*right*. The cells are electrically paced, mechanical stretch is initiated at t=1.2sec, 60msec after the electrical stimulus (Δt_{EM} /$T_{contraction}$=0.27). 20 min. after continuous cyclic mechanical loading, the cell contracts 450 milliseconds before the electrical stimulus (τ_E/$T_{contraction}$=1.6). A second contraction is generated following the electrical stimulus. The movie is played in real time. Scale bar is 25μm.

Movie S4: Bead displacements generated by beating cardiac cells after 1 minute of cyclic stretch applied 25msec before the electrical stimulus (Δt_{EM} /$T_{contraction}$= -0.1) Time lapse imaging of the deformation field generated by a cardiac cell on a flexible substrate. Mechanical deformations are measured by monitoring the position of 0.2μm fluorescent beads embedded within the gel. The corresponding graph for the normalized strain generated by the beating cells as a function of time is shown in Figure 4C-*middle*. The cells are electrically paced, mechanical stretch is initiated at t=0.42sec, 25msec before the electrical stimulus (Δt_{EM} /$T_{contraction}$= -0.1). No change is observed in the beating profile of the cardiac cell. The movie is played in real time. Scale bar is 25μm.

Movie S5: Bead displacements generated by beating cardiac cells after 20 minute of cyclic stretch applied 25msec before the electrical stimulus (Δt_{EM} /$T_{contraction}$= -0.1) Time lapse imaging of the deformation field generated by a cardiac cell on a flexible substrate. Mechanical deformations are measured by monitoring the position of 0.2μm fluorescent beads embedded within the gel. The corresponding graph for the normalized strain generated by the beating cells as a function of time is shown in Figure 4C-*right*. The cells are electrically paced, mechanical stretch is initiated at t=10.44sec, 25msec before the electrical stimulus (Δt_{EM} /$T_{contraction}$= -0.1). 20 min. after continuous cyclic mechanical loading, the cell contracts 240 milliseconds before the electrical stimulus (τ_E/$T_{contraction}$=1.25). The movie is played in real time. Scale bar is 25μm.

Movie S6: Bead displacements generated by beating cardiac cells after 20 minute of cyclic stretch applied 130msec after the electrical stimulus (Δt_{EM} /$T_{contraction}$= 0.91) Time lapse imaging of the deformation field generated by a cardiac cell on a flexible substrate. Mechanical deformations are measured by monitoring the position of 0.2μm fluorescent beads embedded within the gel. The corresponding graph for the normalized strain generated by the beating cell as a function of time is shown in Figure 5C. The cells are electrically paced, mechanical stretch is initiated at t=0.5sec, 130msec after the electrical stimulus (Δt_{EM} /$T_{contraction}$= 0.91). 20 min. after continuous cyclic mechanical loading, the cell contracts on average 265 milliseconds before the electrical stimulus ($\tau_E/T_{contraction}$=1.86). The distribution of time shifts monitored during this movie is shown in Figure 4F. The movie is played in real time. Scale bar is 25μm.

Movie S7: Calcium transients generated by beating cardiac cells under electrical field stimulation Time lapse imaging of the calcium transient generated by electrically paced cardiac cells on a flexible substrate. Cardiac cells were transduced with rAV^{CMV}-GCaMP5, a genetically encoded calcium indicator. The corresponding graph for the calcium signal is shown in Figure S3A. The movie is played in real time. Scale bar is 25μm.

Movie S8: Simultaneous imaging of bead displacement and calcium transients generated by beating cardiac cells paced electrically. Simultaneous time lapse imaging of the calcium transient (right) and bead displacement field (left) generated by beating cardiac cells on a flexible substrate. Cardiac cells were transduced with rAV^{CMV}-GCaMP5, a genetically encoded calcium indicator. Mechanical deformations are measured by monitoring the position of 0.2μm fluorescent beads embedded within the gel. The cells are electrically paced, mechanical stretch is initiated at t=2.52sec,

120msec after the electrical stimulus (Δt_{EM} /$T_{contraction}$= 0.62). The corresponding graph for the calcium signal is shown in Figure S3B. The movie is played in real time. Scale bar is 25μm.

Movie S9: Beating of actin-labeled cardiomyocytes Time lapse imaging of actin labeled cardiomyocytes. Cardiac cells were transduced with rAV^{CMV}-LifeAct-RFP. A snapshot from the movie is shown in Figure S5. The movie is played in real time. Scale bar is 10μm.
